# Food Science Challenge: Translating the Dietary Guidelines for Americans to Bring About Real Behavior Change

**DOI:** 10.1111/j.1750-3841.2010.01973.x

**Published:** 2011-01

**Authors:** Sylvia Rowe, Nick Alexander, Nelson Almeida, Richard Black, Robbie Burns, Laina Bush, Patricia Crawford, Nancy Keim, Penny Kris-Etherton, Connie Weaver

**Keywords:** dietary guidelines, food scientists, nutrition

## Abstract

Food scientists and nutrition scientists (dietitians and nutrition communicators) are tasked with creating strategies to more closely align the American food supply and the public's diet with the Dietary Guidelines for Americans (DGA). This paper is the result of 2 expert dialogues to address this mandate, which were held in Chicago, Illinois, and Washington, D.C., in early October 2010 between these 2 key scientific audiences. It is an objective that has largely eluded public health experts over the past several decades. This document takes the perspective of food scientists who are tasked with making positive modifications to the food supply, both in innovating and reformulating food products, to respond to both the DGA recommendations, and to consumer desires, needs, and choices. The paper is one of two to emerge from those October 2010 discussions; the other article focuses on the work of dietitians and nutrition communicators in effecting positive dietary change.

## Setting the Stage

The Dietary Guidelines for Americans (DGA) were first formally introduced to the public in 1980, in an attempt to give consumers the science-based nutrition recommendations they need to build a healthy diet and prevent diet-related chronic disease. In the intervening decades since 1980, dietary lifestyles have not noticeably improved in the United States. Moreover, so-called lifestyle diseases, including diabetes, heart disease, cancer, osteoporosis, and especially obesity, have become more prevalent in the population, with dramatic increases in some conditions such as obesity and overweight. Although dietary guidance has become increasingly science based, there seems to be an ever-widening gap between the scientific evidence and consumer behavior. The 2010 Dietary Guidelines Advisory Committee (DGAC) report offers a new approach to making dietary recommendations and sounds a new note of urgency: the 2010 DGA are the first to call for a modification of the *food environment* and there is a new chapter on “translating and integrating the evidence, a call to action.” The role of physical activity as part of the energy balance equation to reach public health goals has also been given a higher priority. The need for translating the evidence into real behavior change has never been greater, as has the need for appropriate communications to the public.

As should be clear after 6 DGA reports in the past 3 decades, dietary change is not easy to achieve. It requires 2 key components to succeed: (1) food scientists working within industry or in academia need to reformulate product offerings or create new food products to help balance food choices available to consumers, and (2) consumers need to be truly motivated by nutrition science around human health. These concepts are similar to a push/pull scenario: industry creates the push by developing foods in line with the DGA and consumers pull by demanding healthier foods or, alternatively, by adopting new behaviors that will drive innovation and product reformulation. This paper focuses on the first component to address how food science can modify the food supply and the realities and challenges that pose barriers to change.

Although Americans describe themselves as being relatively familiar with the DGA (Intl. Food Information Council 2010), this familiarity has yet to translate into meaningful modification of dietary lifestyles (Rowe and Alexander 2009). There is even evidence that Americans are confused by the past 6 iterations of the DGA. A 2010 Centers for Disease Control and Prevention (CDC) report suggests that since the issuance of the 2000 and 2005 DGAs, vegetable consumption has shown zero change and average fruit intake has actually declined—with levels well below DGA targets (CDC 2010). One might surmise that something needs to change. In the words of Linda Van Horn, a professor of preventive medicine at the Northwestern Univ. and chairman of the 2010 DGAC: “What has been done till now isn't working. To do nothing more effective than we have, means that five years from now we'll be in an even worse situation. And that would be unconscionable (Black 2010).”

The challenges are substantial. After so many years of concerted efforts to guide the public to dietary behavior in line with recommendations supported by a growing body of scientific evidence, how do we proceed at this point? In its “call to action,” the DGAC offers an explicit prescription on how we are to proceed:

“A coordinated strategic plan that includes all sectors of society, including individuals, families, educators, communities, physicians and allied health professionals, public health advocates, policy makers, scientists, and small and large businesses (e.g., farmers, agricultural producers, food scientists, food manufacturers, and food retailers of all kinds), should be engaged in the development and ultimate implementation of a plan to help all Americans eat well, be physically active, and maintain good health and function. It is important that any strategic plan is evidence-informed, action-oriented, and focused on changes in systems in these sectors (United States Dept. of Agriculture 2010a).”

The overall communication challenges around the DGA are well known. What is less well known are the challenges around modifying the food supply to aid in dietary behavior change. This paper takes those challenges as its focus in the hope that clarification will build a basis for understanding among all stakeholders in the food chain that are alluded to in the DGAC report.

## A Little History

U.S. government food and dietary guidance began in the early 20th century; food groups were identified and recommendations were issued to the public about how to choose foods from the different groups to achieve a healthy diet. A more rigorous process was initiated in 1977 with the “Dietary Goals for the United States,” which was issued by the U.S. Senate Select Committee on Nutrition and Human Needs. A 1979 White House conference led to the formation of a panel of scientists to study the relationship between diet and health. The panel's report, *Healthy People: the Surgeon General's Report on Health Promotion and Disease Prevention*, was published in 1979, and the first DGA was enacted into law in 1980 (Barrett and Kroger 2000).

Since then, a committee of experts has been convened every 5 y to update the guidance, which is then duly reported to the Dept. of Health and Human Services (DHHS) and the United States Dept. of Agriculture (USDA), the 2 government agencies charged with promulgating the recommendations. Just as regularly as they are updated, the guidelines have been more or less ignored by the public. A recent Natl. Cancer Inst. statistical study of dietary intake patterns in the United States led to the following rather grim, but unsurprising, statement: “In conclusion, nearly the entire U.S. population consumes a diet that is not on par with recommendations. These findings add another piece to the rather disturbing picture that is emerging of a nation's diet in crisis (Krebs-Smith and others 2010).”

In compiling its report, the current DGAC had access to an unprecedented quantity of scientific research devoted to diet, which was collected through the USDA's continually updated Nutrition Evidence Library. The 2010 DGAC report was the first dietary guidance to explicitly recognize the importance of the environment, ubiquity of abundant calorie-dense food, and limited opportunities for physical activity. In calling for a multipartnered approach to make the new guidelines effective, the committee explicitly recognized the complexity of the process. Clearly, along with the communication challenges, there must be changes in the food environment. *T*he food supply needs to be modified such that it makes available healthful choices that are in alignment with the DGA accessible and desirable to the public.

## Prescription for Change

The prescription for change poses a major challenge for food scientists. If food products are renovated or modified in ways that impinge negatively upon consumer perception, the modifications will fail to win consumer acceptance and will have no beneficial effect on dietary behavior. Common examples of this effect offered by food scientists are reduced-sodium products, which typically fail in the market because consumers perceive them as tasting badly (see the Burns roundtable abstract in the Appendices). In addition, there are significant financial barriers to reformulation. For example, expensive items include: product development costs, consumer research, higher cost of alternative ingredients, loss-of-opportunity costs, promotional expenses, and so forth. If food companies are simply to offer new products with healthier nutrient profiles, there are also significant hidden expenses for increased inventories and production inefficiencies, as well as for consumer confusion or lack of acceptance, among other reasons. If food products are fortified with healthier ingredients to better align them with the DGA, there may be unforeseen nutrition challenges. For example, if extra fiber or other plant nutrients are added to some foods, increased phytate levels may cause iron and other mineral absorption issues (see the Almeida roundtable abstract in the Appendices). Some of these concerns can be addressed (Mangels and Messina 2001) through higher intakes of foods containing vitamin C, as well as food preparation techniques such as soaking beans, grains, and seeds; leavening bread; using fermentation processes, and so forth (Craig and others 2009). In addition, there is always the concern when adding beneficial nutrients that the processing required does not adversely affect the desired health benefits. The point is that these are not simple changes, but rather they are often complex and expensive modifications that are uncertain to appeal to consumers—not to mention the communication challenges around such reformulations or innovations.

Underlying all of these challenges is the industry imperative to stay in business. To continue producing the nation's food, the food industry must take consumers to a large extent as they are, complete with desires, needs, and likes and with financial constraints (bearing in mind the importance of price points for food products). Marketing and the funding of continuing operations, including research and development, are critical to the bottom line.

Furthermore, the DGA affords the food industry an opportunity to innovate and reformulate products to accomplish something extra beyond satisfying consumer palates and economic requirements. The DGA affords the industry an opportunity to confer health benefits to consumers and to embrace the role of acting as a *provider*, not simply as a merchant. Other opportunities may also exist because food and packaging technology can improve the quality, taste, and nutrition of packaged food products, and it can also help reach the goals of controlling portion sizes and reducing the environmental footprint.

## Call to Action: A Scientific Synergy

In 2010, a group of 4 science and science communications organizations undertook efforts in response to the DGAC's call to action to create synergy between food scientists and dietetic professionals through a robust scientific dialogue, with the ultimate goal of helping to integrate and translate DGA evidence into true behavior change. The 2 audiences, food scientists and dietitians/nutrition communicators/counselors, are key to achieving synergistic solutions for making dietary guidance effective, thereby reaching public health goals. Food scientists are tasked with innovating and renovating or reformulating food products, whereas nutritionists counsel clients and communicate dietary guidance to the public.

The American Dietetic Assn., Inst. of Food Technologists, Intl. Food Information Council (IFIC), and the North American branch of the Intl. Life Sciences Inst. convened 2 expert roundtables of rigorous discussions, whose purpose was to enable the 2 key scientific audiences to interact, innovate, and close the knowledge gaps that are crucial to integrating and translating the DGA. As stated at the outset, the content of this paper is formed from the proceedings of the roundtables held in early October 2010 in Chicago, Illinois, and in Washington, D.C.

The key DGA audiences—food scientists, dietitians/nutrition communicators, and government representatives—gathered with a select panel of speakers at each event. Guided by the DGAC report's new chapter urging translation and integration of the nutrition evidence, participants presented and critically analyzed ideas raised as the principal action themes of the report:

- Reduce the incidence and prevalence of overweight and obesity in the U.S. population by reducing overall caloric intake;
- Shift food intake patterns to a more plant-based diet that emphasizes vegetables, cooked dry beans and peas, fruits, whole grains, nuts, and seeds; and
- Reduce intake of foods containing added sugars, solid fats (SoFAS), refined grains, and sodium (USDA 2010a).

The 2010 DGAC physical activity recommendations were not specifically addressed because they fell outside of the primary expertise of the participating food scientists/nutritionists. The roundtables, in addition to the speaker panels, had other distinguished participants called “discussants,” whose job was to draw out both speakers and other discussants on the opportunities and challenges in achieving real DGA change. The tone of the dialogues was proactive, and all present were urged to focus on success stories or paths deemed promising in accomplishing DGA and public health goals. Two well-known successes cited were the consumer switch to whole-wheat products and the move away from trans fats. In August of 2010, for the first time, sales of whole-wheat bread products surpassed those of refined wheat breads (Bryson 2010). There has also been a move away from products containing trans fatty acids in recent years. Food industry reformulations, following recommendations in the 2005 DGA that trans fat consumption be as low as possible, have resulted in the substantial replacement of trans fats in the American food supply with mono/poly unsaturated fatty acids (Reinberg 2010).

## The Roundtable Presentations

Each daylong roundtable began with an overview of DGA history, with special attention to the 2005 gaps and successes, and included a review of food supply changes since 2005. In the interest of brevity, abstracts of the presentations will be excerpted in this article. The complete presentation abstracts are included in the Appendices.

### 2005 gaps and successes and changes in the food supply since 2005

Connie Weaver, 2005 DGAC member of the Purdue Univ., spoke of the emphasis in the latest DGAC report on obesity and on the excess of solid fats and added sugars (SoFAS) in typical diets, as well as on the recommendation that Americans shift to a more plant-based diet—more fruits and vegetables, dry beans, low-fat dairy, and less-lean meat than currently consumed. She said that a major challenge will be “to provide foods in affordable quantities that compete with foods rich in SoFAS and sodium for the palate of consumers.”

### Challenges and opportunities for implementation of the 2010 guidelines

Laina Bush, U.S. DHHS, presented results of government research into means of overcoming some of the barriers to compliance with the DGA among certain low-income and ethnic subpopulations. Generally, the research shows that significant barriers are sociocultural, culture-based food preferences, lack of readiness to change dietary behavior, and lack of personal or family preference for fresh fruits and vegetables. Food preparation customs were also cited as barriers to compliance with the DGA, as well as psychological distress and psychosocial stress. In addition, the high cost or perceived high cost of food was the most often cited barrier for 3 of the 5 groups studied.

Joe Derochowski of the NPD Group, a global research firm dealing in consumer behavior and retail sales and marketing data, offered his take on consumer behavior in the face of dietary guidance. One of the hallmarks of American society's evolution over the past few decades has been the entrance of women, en masse, into the workforce. That event has altered American lifestyles in a major way: convenience rules, with its prepackaged meals, ready-to-eat food products, fast food, and casual restaurants as replacements for home-prepared meals, and the other trappings of our busy, multitask-laden, 24/7 modern lives. Cooking is becoming a largely forgotten skill.

“The growing need for convenience has given rise to quickly prepared meals, like frozen and ready-to-eat; appliances that enable food to be prepared quickly or with little or no effort; and drive-thru windows. While the percent of women working appears to have reached its peak since 2000, convenience remains at the center of this country's day-to-day lifestyles.”

Derochowski stated that the fundamentals of marketing apply also to the DGA. Food scientists and dietitians need to make it “convenient” for the consumer and for mom to integrate the DGA in the midst of all of their responsibilities. This includes the nature of each of the meal occasions—breakfast being about health, routine, and mobility; lunch being about speed; and dinner being about convenience.

“Mom is the key to fully integrating the Dietary Guidelines into her family's lifestyle. In order for her to accomplish this, we need to make it easy and seamless for her. The guidelines need to become part of the daily routine, quick and convenient to apply throughout the day, everyday. Since home is the primary source of meals, how can she easily implement the Dietary Guidelines into meal planning for her family?”

### The path forward: addressing 3 key concepts of the 2010 DGAC report

#### “Reduce the incidence and prevalence of overweight and obesity in the U.S. population by reducing overall caloric intake.”

Patricia Crawford, DrPH, Univ. of California at Berkeley, is a child obesity specialist and offered the group her view that the best strategy for curbing the prevalence of childhood obesity is to tackle the prevention of it, that is, the incidence—with education and behavior change as well as a healthy food environment, even among the youngest consumers. She pointed out that the 2010 DGAC, for the first time, examined the impact of the food environment on dietary intake and body weight—the environmental focus included restaurants, especially fast food restaurants, portion sizes both at home and away, food access at schools, and supermarket location, particularly as it relates to access to nutrient-dense foods.

Richard Black, PhD, a nutrition scientist at Kraft Foods, offered the group some of his company's insights about the obesity issue:

“It has been proposed that, ‘self-monitoring, including knowing one's own calorie requirement and the calorie content of foods, helps make individuals conscious of what, when, and how much they eat,’ can be an effective approach to limit excessive energy intake (USDA 2010a).”

“…the large majority of the American public is unaware of its own caloric needs, and is equally naïve about the caloric content of most foods (IFIC 2010). Furthermore, daily variances in activity level compound the issue insofar as people are generally unable to estimate energy expenditure for most daily tasks, and for more intense bouts of exercise. And while it remains true that with a great deal of guidance (often from a registered Dietitian), or with the help of a commercial computer program, individuals may have some success in gauging their energy needs and their energy intake, counting calories is a tedious behavior and difficult to maintain in the absence of intense support.”

“While simply ‘selling fewer calories’ might seem the most obvious approach for the food industry, this is not a viable approach. As a thought experiment, consider the following: Company A decides to reduce the calories in its beverage product by 25%, and so aim to ‘sell fewer calories.’ However, when the public becomes aware of this lower calorie version of the drink, demand for the drink increases, and within a year, sales have increased by 35%. In effect, the Company A is now selling 10% more calories as a result of the calorie reduction. However, if consumers have switched from other higher calorie beverages to consume this lower calorie beverage, then the total calories sold by the food industry would be reduced.”

“Portion control is another successful approach to reduce energy intake. In this case, the food industry is ideally situated to provide products in portion-controlled servings, though consideration must be given to the potential for increased packaging and its environmental impact.”

“Interestingly, some efforts undertaken by the food industry to significantly reduce intake of calories and saturated fats seem to be dismissed as insufficient in the report of the DGAC. To whit, when a natural cheese is made from 2% dairy, it can be up to 45% reduced in saturated fat and over 30% reduced in calories. Similarly a slice of American processed cheese can be up to 50% reduced in saturated fat, and 30% reduced in calories. These are certainly behaviorally and health relevant changes, but the DGAC report recommends that Americans consume dairy only when it is either 1% or 0% milk fat. Therefore, 2% cheese is not seen as a viable dietary alternative to full fat cheese, despite the fact that it is palatable, affordable, available, and requires little if any change in diet patterns, whereas cheese made from 1% or 0% dairy is generally reported as unpalatable, somewhat more expensive, and requires significant commitment to incorporate into a diet plan.”

“Clearly, options to modify the diet do exist. However, communications about those options, and people's motivation to utilize those options, remains a challenge. Recommending dietary change that is so extreme as to be only aspirational rather than achievable will not serve the greater public need for dietary guidance to address the obesity epidemic. When all sectors work together, and acknowledge the limitations faced by each, it is possible to imagine the development of a dietary guidance plan that provides options for small changes, which over time can build one upon the next, and gradually lead the American public to healthier and more sustainable eating patterns. After all, we are asking people to fundamentally change how they think about food, shop for food, prepare food, and eat food. This will take time, patience, commitment and trust from everyone.”

#### “Shift food intake patterns to a more plant-based diet that emphasizes vegetables, cooked dry beans and peas, fruits, whole grains, nuts, and seeds.”

Nancy Keim, USDA, Agricultural Research Service, and Lindsay Allen, PhD, USDA, Agricultural Research Service Univ. of California at Davis, collaborated on a presentation: among other points, they suggested that consumer taste likes and dislikes, some of which are genetically based, are a major challenge to vegetable acceptability. Food preparation time is also a major constraint to increasing consumption of dry beans and peas. They had a suggestion for the food scientist participants: if the food industry could process quicker-to-cook forms of dry legumes, consumers might find them attractive.

Nelson Almeida, PhD, FACN, a food scientist with Kellogg Co., summarized a strategic global study of potential industrial earnings for plant-based foods (across 200 markets, there is a projected growth of 40% from 2009 to 2014, with sales potentially increasing from $39.1 to $54.7 billion).

“The Mintel International Group analyzed attitudes towards food in a 2009 internet consumer research study of 2,000 demographically representative U.S. adults aged 18+, and Experian Consumer Research data from July 2007 to September 2005. Given the sharp rise in overweight Americans and various government, media and food company efforts to slow, stop or reverse this trend, consumers are more aware of their diet and the link of diet to health. 86% of U.S. adults said that healthy eating is very or somewhat important to them. Consumers of meat and dairy substitutes wish to improve their nutrition and health, manage their risk for heart disease, lose weight, and increase the safety of the food they eat…”

“Currently, the wheat flour tortilla is the fastest growing product line of all grain-based products. This might be indicative of a growing interest in … whole grains and/or fiber intake for health. Plant-based meals that contain more fiber and consumption of higher fiber plant foods over all meals throughout the day also allow for a higher intake of vitamins, minerals and bioactive phytochemicals, as well as promotion of satiety, intestinal health and reduction of chronic disease risk…”

“It is the complement of the wide array of whole grains, fiber-based products, legumes, fruits, vegetables, nuts and seeds that makes a plant-based diet appealing, nourishing, tasty and healthful. Additions of fiber and bioactive portions of plants to the current set of food offerings is an important goal for food company research and development. It is that endless combination of predominantly plant-based meal selections made with simple, single ingredient foods as well as processed multi-ingredient foods that allow for balance, variety and moderation throughout the day…”

“Desserts can also provide more plant-based appeal with inclusion of more fruit in the more traditional product offerings, like fruit streusels, but also with fruit as fat replacers, and additives such as dried fruit in cookies, muffins and quick-breads.”

“As Americans become better informed about healthy food combinations and practice these learnings, plant-based foods can become a greater part of the diet. As children and adolescents learn more about plant foods and plant-based diets through educational efforts in schools including cooking classes and community-based gardens with better communication on the Internet and advertising, it is hoped that future generations have a better understanding of food's connection to health, disease risk-prevention and well-being.”

#### “Reduce intake of foods containing added sugars, solid fats, refined grains, and sodium.”

Penny Kris-Etherton, PhD, The Pennsylvania State Univ., sees the gap between dietary recommendations and current consumer behavior along with heightened interest in diet and health as an opportunity to develop diet-improving interventions. She told participants that on an individual and group basis, cognitive-behavioral strategies have proved effective in behavior change—notable among these is motivational interviewing, with its well-ordered feedback and monitoring.

Robbie Burns, formerly of Cadbury, represented the food scientist perspective on this issue and pointed out that “time delays between changes in dietary intake and health outcomes confound the ability to draw strong conclusions about the overall health impact of the DGA.”

“Since their inception in 1980, Dietary Guidelines for Americans (DGA) have included, in some form, advice to decrease dietary intakes of added sugars, solid fats, refined grains and sodium. As a result and to meet consumer desires for more healthful products, the food industry has developed alternatives where all these negative components are reduced and in some cases eliminated.”

“In order to remain viable, food manufacturers must make products that meet consumer desires for taste, price and convenience (portable, easy to prepare, etc.; IFIC Food and Health Survey 2010). In most product categories, health aspects of the food have a lower priority than taste or price. Much of the progress towards meeting the DGA has been through decreasing saturated fat, trans fat, refined grains and/or sodium in ways that are not obvious to the consumer. However, some of the positive changes in the composition of food have been offset by increased food consumption.”

“The 2010 DGAC recommendations includes some notable changes from those of 2005; the goal for dietary saturated fat is decreased from less than 10% to less than 7% of energy; cholesterol targets are decreased from less than 300 mg/day to less than 200 mg/day and the sodium target is decreased from less than 2300 mg/day to less than 1500 mg/day. Simply raising the goal is unlikely to effect a greater rate of change in dietary habits unless accompanied by better ways to induce long-term changes in consumer behavior and/or specific engagement by food manufacturers in education efforts, creation of new products (e.g. to shift intake patterns; addressed elsewhere in this workshop) and/or reformulations of existing products.”

“Education efforts include information on websites and product packaging to highlight positive health messages such as the food pyramid. Food manufacturers can also modify marketing strategies to focus promotions on products that more closely adhere to the DGAC recommendations; e.g. by changing the relative advertising spend on diet or low-calorie beverages compared to sugar-sweetened beverages…”

“Product reformulations within the realms of available technologies will not be sufficient to achieve the DGAC recommendations without changes in the pattern of foods consumed. However, for many foods that contribute significant quantities of added sugars, solid fats, refined grains, and sodium, reformulations can help meet goals when applied judiciously over a period of time. Note that it has taken over 20 years for consumers to change from full fat milk to lower fat options.”

“Food technology continues to provide tools to enable manufacturers to reduce undesirable components in certain foods. In some cases, the component can be replaced by a more desirable component such as polyunsaturated fat for solid fat and whole grain for refined grain. However, in other instances food additives are required. In these situations it is essential that the product not only meets consumer taste preferences, but also addresses their desire for simplicity and, in some cases, their fears of novel technologies.”

## Strategic Priorities

The roundtable discussions each day focused on the real-world challenges and opportunities of product innovation/renovation as well as education and communication in implementing the guidelines. Participants raised several main themes that were echoed and elaborated on throughout the sessions, as described below.

- There needs to be a coordinated strategic plan with the active involvement of all sectors to achieve effective implementation.
- Trust is a key factor in communicating dietary goals and modifying the food supply; mutual understanding and trust among all stakeholders is critical to implementation and requires input from industry and academic food scientists; public health professionals; dietitians, nutrition communicators, and counselors; and others in the food chain, as well as government.
- The DGA should be viewed as aspirational with the bar set high. If the guidelines are seen as all-or-nothing goals, there would be no room to embrace or celebrate small changes and incremental dietary progress.
- Consumer messages around nutrition and especially weight loss need to be even simpler and more targeted than the past communications of the DGA.
- Sociocultural factors should be a major consideration in composing messages for consumers of different ethnic and demographic groups.
- In considering the best evidence for what works and what does not work to improve consumer dietary choice, the best available evidence should point the way.
- Children's nutrition education seems an optimal starting point for changing adult dietary patterns.
- Behavioral science needs to be employed in drafting messages to influence consumer dietary choices (USDA 2010b).
- Environmental modifications need to be part of any overall strategy in altering dietary patterns and need to be reinforced in messaging so as to enable the healthy choice to be the easy choice (Yach 2002).
- Food scientists, in striving to innovate and reformulate products, should employ both gradual modifications, or “stealth” methods, where consumers would perceive no change whatsoever in their favorite foods as they became healthier, and also education, or transparent means, whereby consumers would be encouraged to understand healthful modifications in their food.
- There is a need to communicate more fully to the public the complexities inherent in enhancing nutrient profiles of their accustomed foods.

Although the DGAC raised the issue of sustainability in its report, the roundtable participants acknowledged the issue but did not address it explicitly. Similarly, although the roundtable participants discussed the environmental focus of the DGAC, this topic was not addressed in depth. The broad thematic ideas listed above inspired a rich discussion in both roundtables, with participants producing a variety of ideas, suggestions, and insights that will be highlighted in the following paragraphs. Within these somewhat broad thematic concepts, there were numerous points offered in greater specificity.

### A coordinated strategic plan for all sectors

Food scientists working with industry emphatically made the point that there has been a lack of trust among the various stakeholders in the food chain and this must change if the country is to achieve a sustained dietary behavior change. To quote the DGAC, “A coordinated strategic plan that includes all sectors of society, including individuals, families, educators, communities, physicians and allied health professionals, public health advocates, policy makers, scientists, and small and large businesses (for example, farmers, agricultural producers, food scientists, food manufacturers, and food retailers of all kinds), should be engaged in the development and ultimate implementation of a plan to help all Americans eat well, be physically active, and maintain good health and function” (USDA 2010a). Participants in both roundtables overwhelmingly concurred, and some suggesting adding stakeholders that had not been listed by DGAC such as behavioral scientists and consumer advocates.

### Trust and mutual understanding

An often-repeated refrain in both roundtables was that: “Public health advocates don't trust industry; industry does n't trust government; there is too much mistrust currently for all interested groups to work together effectively.” One food scientist participant pointed out that some policy-making scientific groups specifically prohibit industry-employed scientists from advisory panels, which is a prohibition he said he did not understand because there is an opportunity to provide industry expertise on such panels to widen and deepen the scientific knowledge base being drawn upon. Bringing together industry and academic scientists in a common public health cause would also create a growing basis for trust between food scientists and public health professionals. One approach might be to establish collaborative research teams of nutrition and food scientists to document that health benefits are still attained after the “form” of the nutrient component has been changed to meet food production and consumer preferences. Roundtable participants pointed out that an excellent example of trust building was provided in the discussions and sharing of viewpoints during the roundtables themselves. Food scientists and dietitians/nutrition educators/counselors were able to forge mutual understanding of their different perspectives as they strategized together around implementing the DGA. One major conclusion at both the Chicago and Washington meetings was the enormous, positive potential of collaboration: if all stakeholders can be helped to understand the demands on other players in the space and work together, they can attack the challenges from many angles, employing different approaches to achieve the same end.

### Aspirational goals, but reachable incrementally

There was considerable discussion in both roundtables about taking a longer term view toward dietary change by taking account of intermediate dietary successes, rather than an all-or-nothing view. Industry food scientists pointed out that their companies had already reformulated a number of products to better align with the DGA, and much work along those lines is still being done. They suggested that given the key requirement of market acceptance, it was perhaps impractical to expect immediate and radical changes in dietary behavior. Intermediate dietary objectives and opportunities for interim behavior modification might be a more productive way to achieve broader public health goals. The incremental approach enables consumers to celebrate small improvements and motivates them to “stay the course” for greater change. As Voltaire observed in the 18th century, “the perfect is the enemy of good.” Many roundtable participants agreed with this view; some made the point that reevaluating currently employed language to communicate nutrition messages with an eye toward simplification might also be productive. It was also suggested that dietary recommendations should not only be simple and realistic, but also few in number, with no more than 3 overarching messages at a time. Participants of both roundtables agreed that the public health community would benefit by promoting small steps in behavior change and developing ways to measure incremental dietary improvements. They also agreed that although product reformulations could achieve and are, in fact, already achieving small changes in consumer behavior, more can and still needs to be done.

### Taking account of culture

Food scientists and others who communicate to consumers should take sociocultural factors into account when innovating/renovating products and composing messages. It is well known that different subpopulations have different taste preferences and dietary customs. Some participants pointed out that because of deep-rooted sociocultural traditions, shifting people's eating habits is extremely difficult. It has been noted that ethnic groups will often pay more for food with which they are familiar than for recommended healthier alternatives. Food scientists will be constrained in their development work by some of these attitudes; however, there is also an opportunity to tailor products, marketing, and nutrition messages to specific consumer groups. Considering the Hispanic American subpopulation, one participant pointed out that in communicating with these subpopulations, the messenger may be as important as the message (for example, *abuelas* or grandmothers, who are trusted advisers in food and health matters among Hispanic populations). The most effective dietary change strategies should be highly targeted dietary messages that are geographically and socioculturally targeted, and messages that do not link physical activity and dietary changes. It was also acknowledged that sociocultural conditions and consumer habits are realities for all populations, not just ethnic or underserved subpopulations.

### Dietary behavior patterns are set early in life

Although obesity in general and childhood overweight in particular are among the most challenging issues raised in the 2010 DGAC, roundtable participants believe that there are some key opportunities for addressing obesity. Discussants made related points, suggesting that some of the most successful behavior-modification campaigns have started with school-age children (recycling, seatbelt safety, antismoking, and so on). One participant argued that in a highly structured environment, such as a calorie-regulated summer camp, research shows that given a healthy food and activity environment, “every biomarker for children's health improves.”

Participants observed that nutrition education at the very earliest educational stages could yield the most profound results, especially when linked with a healthy food and activity environment. By also targeting the parents of young children with consistent dietary-behavior messages, there may also be a chance to reach children through their parents. One participant made the point that involving mothers in children's education outreach is critical, and that any education of the child should be transferable to his or her mother either through sharing learning materials or inviting mothers to participate in their children's education.

Another discussant referred to a new cell phone “app” that is capable of tracking energy balance, stating that such devices could well help educate children through their “cool factor.”

Because celebrity chefs have become popular invitees into schools, a similar opportunity may exist for dietitians and nutrition educators to follow suit. There was wide agreement among participants to support the DGAC's recommendation to stress cooking skills, both in family settings and in schools.

## Using Strategies That Work

Along the lines of messaging, some participants drew on an Inst. of Medicine (2007) recommendation that when definitive research conclusions are not available, policy makers should work with the best evidence available, instead of delaying policy. The roundtable participants agreed that using the best available evidence about what works and what does not work should be the basis for action, while recognizing that the American public cannot be fitted with a one-size-fits-all dietary strategy. This is an opportunity for dietitians, nutrition communicators, and counselors to personalize guidance based on the needs and desires of individual consumers.

### Calling psychologists and behavioral economists

Roundtable participants argued on multiple occasions that behavioral science must be part of the dietary change process, both in modifying preferences and in drafting messages to influence consumer dietary choices. Some of this work has already begun at the USDA (2010b). There was considerable discussion of cognitive-behavioral strategies in improving consumer diets, although these tend to be highly labor intensive and expensive. Food scientists formulating new and existing products already employ strategies keyed to consumer preferences (see the discussion of “stealth” modifications in the next paragraph). Industry representatives among the roundtable participants made the point that although products can easily be launched, they will not succeed in the marketplace without consumer acceptance, which involves meeting consumer expectations of taste, convenience, and cost.

### Stealth versus persuasion

Consumers have been resistant to dietary change, partly because of established food preferences: “stealth” methods of change are potentially effective because they do not upset those established preferences. There are existing examples of food products that have been successfully modified to have a healthier nutrition profile without consumers' perception of any change. The highest-profile example is the industry-wide conversion from trans fatty acids to mono- and polyunsaturated fats in product formulations, as noted earlier in this article. And of course, food innovations or reformulations would still be transparent through the product labels, both on the ingredient listing and the nutrition facts panel. Food scientist participants of the roundtables informed their dietitian colleagues that food companies typically spend 60% to 70% of their research and development budgets on renovation and only 30% to 40% on innovation of new food products. Reformulations are therefore the most efficient way to produce more healthful foods.

**Figure 1:**
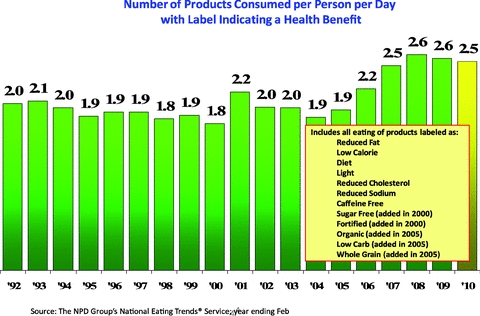
Consumer Healthy Eating Trends 1992 to 2010 (proprietary marketing research, NPD Group [http://www.npdgroup.com]).

Discussants made the point that consumer resistance to dietary change may be an opportunity for both the dietetic and food science communities. As indicated earlier in this paper, dietary change could be effected on a push/pull basis, with nutrition communicators persuading consumers to demand foods in line with the DGA and food scientists innovating and reformulating products to meet that demand and create a marketplace of healthier alternatives.

## Changing Nutrient Profiles: No Simple Task

Roundtable participants heard that 80% to 90% of new food products fail to achieve market acceptance. Some dietary change advocates have argued that the food industry, with its highly persuasive and well-financed marketing departments, can simply produce products with healthier nutrition profiles and then “sell them” to consumers. The 80% to 90% failure rate of new products is a sobering reality check. Both roundtable groups reached a similar conclusion: there is a knowledge gap between the production of food and the selection and consumption of food. Consumers could better understand the challenges of food manufacturing both in terms of reformulation of products without changing taste, appearance, and cost, and in terms of aligning products with the DGA. A public preference for extremely short ingredient lists on processed food products also poses major challenges to food scientists in renovating/reformulating food.

## About the Environment

The 2010 DGAC raised a new issue in its report: the food-related physical environment, from the ubiquity of restaurants and food markets to the “built environment,” which limits opportunities for physical activity. Roundtable participants considered the environment a key consideration, and urged all stakeholders to develop strategies recommending modification of environmental barriers to adopting the DGA. They stressed that in the absence of such strategies, efforts to effectively translate the DGA into behavior change are unlikely to succeed. In the words of a World Health Organization report, the goal should be to enable the healthy food choice to be the easy choice (Yach 2002).

## Consensus Findings

The most important consensus findings from the 2 roundtables, and those themes enjoying the greatest consensus, were as follows:

- There is a critical need for a coordinated strategic plan with the active involvement of all sectors, including industry, academia, public health professionals, and government, to achieve effective implementation of the DGA.
- Both goals—modification of the food supply and more impactful communication strategies—are critical to achieving desired public health outcomes.
- In order for the DGA to have its maximum impact on public health, new approaches need to be employed, such as setting strategic priorities, realistic public health objectives, and placing greater emphasis on practical solutions (for example, food product renovations to achieve incrementally changed dietary behavior).
- Behavioral science needs to be brought to bear on the challenges.
- Dietary messages need to be positive, very simple, few in number, and targeted to subpopulations, both to inform and motivate consumers.
- Messages need to take account of sociocultural factors, consumer habits, and the realities of today's lifestyles.
- To address the obesity epidemic, a key focus should be on very young children and on their parents.
- Above all, trust and collaboration are essential among all stakeholders, including food industry scientists, public health community representatives, government agencies, nutrition communicators, retail food industry organizations, environmental planners, and others.

Perhaps an overarching theme of the roundtables, not adequately captured in the above list, was “practicality.” Those interested in dietary behavior change need to be practical about it by accepting intermediate successes and incremental gains, being patient with change (one food industry scientist pointed out that it has taken more than 20 y for consumers to accept lower fat dairy products [see the Burns abstract in the Appendices]), and combining food reformulations with consumer education (because both strategies are more successful when combined). Patience with making these changes is important from the food scientist's perspective. One participant pointed out that substituting noncaloric sweeteners for sugar in products can be moderately successfull in reducing consumer intake of added sugars, but there have been consumer acceptance issues with “artificial” sweeteners. Furthermore, “natural” sweeteners, such as Stevia with its bitter aftertaste, may require further additives to gain widespread consumer acceptance. And then there is also the issue of consumer preference for few additives. Similarly, lower fat reformulations may work well in some products, but not all. One scientist observed that: “in products such as cheese and chocolate the physical and sensorial properties of the saturated fats are such that significant reductions are not acceptable to consumers.” (See the Burns abstract in the Appendices.) In addition, standards of identity for some products, such as certain cheeses, limit reformulation.

Patience is a key virtue in guarding against excessive expectations, and food industry scientists need to be wary of unintended consequences. In the world of consumers where choice is the currency, in most product categories, the health aspects of the food have a lower priority than taste or price. Patience and perseverance are clearly virtues as stakeholders pool their efforts and abilities to bring the food supply more in line with the DGA. Regarding messaging, the following question was brought up at the roundtables and bears some further thought: “Is there some way to articulate the advantages of good nutrition than simply saying it is ‘for good health’?”

The last point in the above list of roundtable conclusions was clearly the one garnering greatest consensus and was regarded by participants at both roundtables as an essential component of any communication and dietary behavior change program. For a host of reasons, mutual trust and understanding of complementary roles and responsibilities have eroded over the years and need to be rebuilt. Understanding between dietitians/nutrition communicators/counselors and food scientists is decidedly crucial to restoring trust, and collaboration between these 2 groups is critical to pursuing the dietary changes necessary to reach public health goals. Small successes, small steps, and open, transparent processes will do much to build trust among the multiplicity of stakeholders critically interested in seeing Americans' health and dietary regimes reach desired DGAC-recommended goals.

## Appendix

The following individuals attended the roundtables.

### Speakers/Authors

Nick Alexander, SR Strategy LLC; Nelson G. Almeida, Kellogg Co.; Richard Black, Kraft Foods Inc.; Robbie Burns, Nutrition Implications, LLC; Laina Bush, DHHS; Patricia Crawford, Univ. of California, Berkeley; Joe Derochowski, NPD Group; Nancy Keim, USDA Agricultural Research Service; Penny Kris-Etherton, The Pennsylvania State Univ.; Sylvia Rowe, SR Strategy LLC; and Connie Weaver, Purdue Univ.

### Discussants

Jeanne Blankenship, American Dietetic Assn.; Dondeena Bradley, PepsiCo Inc.; Mary Christ-Erwin, Porter Novelli; Janet Collins, DuPont; Suzie Crockett, General Mills Inc.; Johanna Dwyer, Natl. Inst. of Health; Robert Earl, The Coca-Cola Co.; Cecilia Fileti, Latino Health Communications; Constance Geiger, Geiger & Associates; Marianne Gillette, McCormick & Co., Inc.; Jeanne Goldberg, Tufts Univ.; Cathy Adams Hutt, RdR Solutions; Barbara Ivens, ConAgra Foods, Inc.; Guy Johnson, McCormick Science Inst.; Michelle Matto, Intl. Dairy Foods Assn.; Kathy McMahon, Sara Lee Corp.; Melissa Musiker, Grocery Manufacturers Assn.; Jill Nicholls, Natl. Dairy Council; Susan Nitzke, Univ. of Wisconsin; Jessie Pavlinac, Oregon Health & Science Univ.; Mary Pat Raimondi, American Dietetic Assn.; Judith Rodriguez, Univ. of North Florida; Leila Saldanha, NutrIQ LLC; Marilyn Schorin, Schorin Strategies; and Pamela Starke-Reed, Natl. Inst. of Health.

### Observers

Carole Davis, USDA Center for Nutrition Policy and Promotion; Kathryn McMurry, HHS Office of Public Health and Science; and Rob Post, USDA Center for Nutrition Policy and Promotion.

### Staff

Will Fisher, Inst. of Food Technologists; Eric Hentges, ILSI North America; Esther Myers, American Dietetic Assn.; Sarah Ohlhorst, Inst. of Food Technologists; Wendy Reinhardt Kapsak, IFIC; Marianne Smith-Edge, IFIC; Lisa Spence, American Dietetic Assn.; and Heather Steele, ILSI North America.

This paper is the product of a series of roundtables held in October 2010 on translating and integrating the science in the 2010 Dietary Guidelines. The roundtables focused on the opportunities and challenges for 2 key audiences: dietitians and food scientists. This was a collaborative effort between the American Dietetic Assn., the Inst. of Food Technologists, the IFIC, and the North American branch of the Intl. Life Sciences Inst.

## Supporting Information

The following supporting information is available for this article.

Abstracts of Roundtable Speaker Presentations are available at http://www.ilsi.org/NorthAmerica.
